# Adverse Outcomes of Patients with Non-Ventilator-Associated Hospital-Acquired Pneumonia (nvHAP)—A Single Centre Cohort Study

**DOI:** 10.3390/idr16020018

**Published:** 2024-03-13

**Authors:** Enrica Amodio, Peter W. Schreiber, Mirjam Faes Hesse, Aline Wolfensberger

**Affiliations:** Department for Infectious Diseases and Hospital Epidemiology, University Hospital Zurich, University of Zurich, 8091 Zurich, Switzerland

**Keywords:** non-ventilator-associated hospital-acquired pneumonia, nosocomial pneumonia, risk factors, outcomes, in-hospital mortality

## Abstract

Non-ventilator associated hospital-acquired pneumonia (nvHAP) is a common nosocomial infection, but little is known about the outcomes of patients with nvHAP and the risk factors for adverse outcomes. In this retrospective study conducted in a Swiss tertiary care centre, adverse outcomes like in-hospital mortality, intensive care unit (ICU) admission, and mechanical ventilation, both all-cause and nvHAP-associated, were investigated. Of 244 patients with nvHAP, 72 (30%) died, 35 (14%) deaths were attributed to nvHAP. While 36 (15%) patients acquired nvHAP on the ICU, another 173 patients were eligible for ICU-transferral, and 76 (43.9%) needed ICU-admission. Of all patients hospitalized on the ICU 58 (51.8%) needed intubation due to nvHAP. Multivariable logistic regression analysis identified lower body mass index (OR per unit increase: 0.90, 95%CI: 0.82–0.98) and lower haemoglobin on admission (OR per unit in g/l increase: 0.98, 95%CI: 0.97–1.00) as patient specific factors independently associated with nvHAP-associated mortality. Given the frequency of nvHAP adverse outcomes, hospitals should evaluate increasing nvHAP prevention efforts, especially for patients at high risk for nvHAP mortality. To what extent pneumonia prevention interventions do lower nvHAP mortality in these patients is still to be evaluated.

## 1. Introduction

Healthcare-associated infections (HAI) are a common adverse event in hospitalized patients. Seven of 100 hospitalised patients in high-income countries, or 15 patients in middle to low-income countries acquire an HAI during their hospital stay [1]. As most point prevalence surveys, the large European point prevalence study from the years 2016/2017 identified respiratory tract infections to be the most common HAI, and among them hospital-acquired pneumonia (HAP) represent the vast majority (21.4% pneumonia and 4.5% other lower respiratory tract infections) [2]. Of all HAP, about two thirds are non-ventilator-associated HAP (nvHAP) [2,3]. At the University hospital Zurich (USZ), a tertiary teaching hospital in Switzerland, the overall incidence rate of nvHAP was reported to be 0.83/1000 patient days in 2017 [4]. 

According to the WHO, crude mortality rates associated with HAI vary from 12% to 80% depending on the study population [5]. Overall mortality rates of around 30% have been reported in patients with nvHAP [6,7], but little is known about how many of the deaths are attributable to nvHAP. Admission to an intensive care unit (ICU) and the need for mechanical ventilation seem to be more likely in patients with nvHAP [8], but data about the causal relation to nvHAP remain scarce. More exact numbers could help to quantify the relevance of nvHAP. Additionally, as nvHAP prevention efforts are especially important in a patient population at high risk for severe disease, data about patient specific factors associated with adverse nvHAP outcomes such as death, ICU admission or mechanical ventilation are of high interest, as some of these risk factors might be targetable by specific measures. The importance to learn more about the above mentioned aspects of nvHAP was highlighted by experts [9,10]. The main objective of this study was to quantify all-cause and nvHAP-attributable adverse outcomes, i.e., in-hospital mortality, ICU-admission, and need for mechanical ventilation of patients with nvHAP. The secondary objective was to identify patient and pneumonia specific factors for nvHAP-associated adverse outcomes. 

## 2. Methods

### 2.1. Study Design, Setting and Patient Population

This retrospective single-centre cohort study was conducted at the University hospital Zurich (USZ), Switzerland, a 900-bed tertiary teaching hospital. All 255 patients with nvHAP from the year 2017 identified by retrospective, semi-automated nvHAP surveillance applying the European Centre for Disease Prevention and Control (ECDC) criteria were eligible [4,11]. This cohort was described in a paper by Wolfensberger et al. [4], and comprised patients admitted to the hospital before the start of a large and successful nvHAP prevention initiative in 2018 [12], which we assumed to have an impact on characteristics of the remaining nvHAP patients. All patients from the year 2017 with nvHAP were included with the exemption of patients who developed first symptoms after discharge (due to missing data). Per definition, patients with nvHAP had symptom onset ≥ 48 h after hospital admission and absence of a respiratory device in the 48 h before symptom onset, with the exemption of short-term respiratory devices due to general anaesthesia. 

### 2.2. Adverse Outcomes and Potential Risk Factors for Adverse Outcome

Adverse nvHAP outcomes were defined as death, ICU admission, and mechanical ventilation, and were classified as either all-cause and nvHAP-associated. In-hospital mortality was defined as death during the current hospitalization. NvHAP-associated mortality was assumed in patients whose respiratory or inflammatory situation did not recover between nvHAP diagnosis and death, and with no other apparent cause of death. ICU-admission after nvHAP was defined as admission to an ICU within 5 days after first symptoms of nvHAP, a causality between nvHAP and ICU admission was assumed in a patient with respiratory failure or any other condition, e.g., sepsis, due to nvHAP. NvHAP-associated intubation was defined as placement of an invasive respiratory assist device after onset of nvHAP and the presence of respiratory failure in relation to nvHAP. Causality of endpoints with nvHAP was evaluated by two main reviewers (EA and AW), in case of disagreement a third reviewer (MFH) was consulted. 

Potential risk factors for adverse nvHAP outcomes were predefined and determined based on existing literature and expert opinion and are listed in Table S1. They included demographic characteristics (age, sex, and body mass index (BMI)), Charlson comorbidity score and it’s elements (Table S2), laboratory parameters, pneumonia specific parameters (pneumonia aetiology, sepsis defined as quick sequential organ failure assessment score (qSOFA) of ≥2 [13], acute respiratory distress syndrome (ARDS), empyema and radiologic presentation of infiltrates). All relevant data was manually extracted from the patient’s electronic medical records (EMR).

### 2.3. Data Analysis

Interrater agreement about causality of nvHAP for all adverse outcomes was assessed using Cohen’s Kappa [14]. Outcome measures (i.e. percent mortality, ICU-admission, and intubation) were analysed descriptively, with percentages calculated of either the total patient cohort, or the patients “at risk” for a certain outcome. That is, patients with a “no-ICU decision” (e.g. due to patient’s will) were excluded from the sub-group analysis evaluating ICU-admission and intubation.

Risk factors for adverse outcomes were identified by univariable and multivariable logistic regression analysis restricted to complete datasets. Variables with a p-value of <0.1 in the univariable analysis and the variable age were included into multivariable analysis. For variables with a high degree of collinearity (i.e., a correlation coefficient of >0.3), one variable was chosen to be entered in the multivariable analysis based on clinical reasoning. Statistical significance was defined as *p*-value < 0.05. Statistical analysis was done in STATA version 16.1 (Stata Corp., College Station, TX, USA).

The STROBE checklist for cohort studies was used as guideline for reporting [15].

## 3. Results

Of all 255 patients with nvHAP from the year 2017, 11 were excluded due to symptom onset after discharge. Of the 244 included, 162 (66%) were men and the median patient age was 67 years (interquartile range (IQR): 55–79), and the median Charlson comorbidity index was 5 (IQR: 4–7) (Table 1). A total of 80 patients were affiliated to internal medicine and subspecialties, 42 to oncology and hematology, 27 to neurology and neurosurgery, 89 to other surgical departments (cardiac, thoracic, visceral, urogenital, or plastic surgery, traumatology), and six patient to other departments. Median length of stay until nvHAP diagnosis was 9 days (IQR: 5–17). The median total length of hospital stay was 26 days (IQR: 16–39.5) (data not shown), longer for patients with (32.5 (IQR: 19–57) than without ICU admission (24 (IQR: 16–37)). Most common co-morbidities were myocardial infarction, cerebrovascular disease, and moderate or severe renal disease.

Table 1 gives an overview about pneumonia characteristics: nvHAP aetiology was proven bacterial in 71 (29.1%) cases, bilateral pulmonal infiltrates were seen in 111 (45.5%) patients, and 79 (32.4%) patients were septic.

### 3.1. Frequency of Adverse Events and Associations with nvHAP

**Figure 1:**
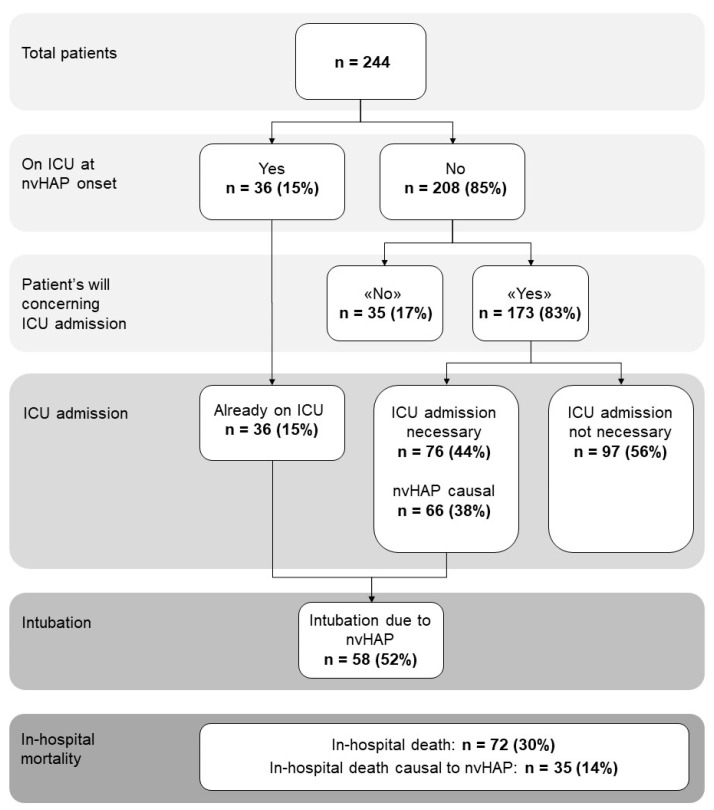
Frequency of adverse events in patient population with nvHAP. Abbreviations: ICU, intensive care unit; n, number; nvHAP, non-ventilator associated hospital acquired pneumonia.

Interrater agreement between the two main reviewers regarding association of nvHAP with the adverse outcome was moderate to substantial (Cohen’s Kappa: 0.72 for association of nvHAP with mortality, 0.67 for association of nvHAP with ICU-admission, and 0.58 for association of nvHAP with intubation). After resolving disagreement in consultation with a third reviewer, results were as follows (Figure 1): during hospitalisation, 72 (30%) patients died, of which 35 (49%) deaths were nvHAP-related. A total of 36 (15%) patients were already on ICU when showing first symptoms of nvHAP. Of the 173 patients on a general ward or intermediate care unit who in principle agreed to ICU transferral, 76 (44%, or 31% of total patient population) needed to be transferred to the ICU, the vast majority due to nvHAP. Intubation due to nvHAP was necessary in 58 patients (i.e., 52% of patients who were on the ICU, or 24% of total patient population). 

### 3.2. Patient- and Pneumonia-Specific Factors Associated with Adverse Events 

Lower BMI and lower haemoglobin on admission were independent risk factors for in-hospital death attributable to nvHAP (Table 2), while bilateral infiltrates and sepsis were pneumonia-specific risk factors (Table 3). 

Patient specific factors associated with all-cause in-hospital mortality in nvHAP patients were a low albumin and a low haemoglobin on admission (Table S3). Patient specific factors associated with ICU-admission due to nvHAP was lower haemoglobin on admission, while pneumonia specific factors were bilateral infiltrates and sepsis (Tables S4 and S5). Patient-specific factors independently associated with the necessity for intubation were low albumin and being of younger age, the only pneumonia-specific factor was sepsis (Tables S6 and S7).

## 4. Discussion

In this retrospective cohort study including all patients from a one year period with nvHAP from a Swiss tertiary care centre, we observed a high all-cause in-hospital mortality of about 30%. We found that about half of the patients (i.e., 14% of the patients with nvHAP) died in temporal and probably causal relation to the nosocomial pneumonia. These results are in line with a study from 1997 including 85 patients with nosocomial pneumonia on general surgical or medical wards, reporting an overall mortality of 20% and an attributable mortality of 14% [17]. A study including 119 patients from 2014 with hospital-acquired pneumonia acquired outside the ICU found a slightly higher overall mortality of 33% and attributable mortality of 28% [7]. Studies investigating all-cause in-hospital mortality of patients with nvHAP found rates of 13% to 31% [6,8,18,19,20]. Compared to ventilator-associated pneumonia, these percentages are somehow lower than the mean 33% VAP-mortality of patients included in a systematic review [21]. Davis et al. and Esperatti et al. directly compared nvHAP and VAP mortality and found 19% vs. 19% in a total hospital patient population, and 36% vs. 42% in ICU patients, respectively [18,22]. However, Kollef et al. found lower mortality rates (19% vs. 30%) in nvHAP than in VAP, notably in a patient population selected by positive microbiology culture [23], and thus probably including more severe nvHAP. Even though mortality is an objective endpoint that is easy to ascertain, the causal relation of nvHAP with death is more difficult to determine. We chose to assess nvHAP associated mortality on a case-by-case basis by in-depth review of the electronic medical records. The substantial interrater agreement between the two reviewers supports the validity of this method in ascribing causal relation of nvHAP to mortality.

In our cohort, 44% of the patients who were not already on the ICU, required ICU-admission. Again, our analysis was executed on individual patient assessment via EMR, excluding patients who were not eligible for ICU-transfer, e.g., due to patient’s will, what precludes an underestimation of the ICU-admission rate. Other authors report diverging results: Mikec et al. found 56% necessity for ICU admission in a cohort pre-selected by including patients who had respiratory cultures obtained, probably representing patients with more critical disease [8]. Others reported lower percentages of 9% and 28% in patients who acquired HAP outside the ICU [7,17]. In our cohort, half of patients admitted to the ICU and a quarter of all patients in total needed intubation. These numbers are supported by most other studies [8,17,22], however, Sopena et al. reported a percentage of patient requiring intubation as low as 6% [7]. These mostly high percentages of ICU admission and intubation underscore the severity of many nvHAP-episodes.

Our analysis identified both patient specific and pneumonia specific factors associated with adverse outcomes, knowing that the latter might not be independent from the former. These risk factors for mortality have so far not been researched thoroughly. Some authors assessed all-cause mortality in nvHAP patients and found that older age [17,24], higher Charlson scores or greater number of underlying diseases [8,17], affiliation to medical ward [17], ICU-admission [24], elevated blood urea nitrogen [24], and lymphopenia [24] to be associated. Several studies investigated HAP in general (including VAP) and moreover identified chronic lung disease [25,26,27], and liver disease as risk factors for all-cause mortality [25,26,27,28]. In our patient cohort with nvHAP, a low albumin and a low haemoglobin on admission were associated with all-cause in-hospital mortality. In comparison, nvHAP-attributable mortality was associated with lower BMI and lower haemoglobin on admission, and boundary associated with older age. It seems that there are not specific comorbidities that put our patients at risk for dying from nvHAP, but more general factors possibly mirroring multimorbidity, depression of the immune system or frailty. Malnutrition for example is associated with depression of the immune system and increased mortality [29], and can be assessed by the nutrition risk screening (NRS) [30], with BMI being one of the parameters in the NRS. Low BMI was also found to be associated with all-cause and infection-related mortality [31]. Similarly, lower haemoglobin is a known risk factor for general mortality [32], and associated with a longer hospital stay and with higher in-patient mortality [33]. In our cohort, lower haemoglobin was also associated with nvHAP-attributable ICU-admission, while age and BMI were not, probably due to reluctance of ICU referral in the oldest patients and patients with unfavourable prognosis.

Bilateral infiltrates were associated with higher mortality, as it was already shown for nursing home acquired pneumonia and for HAP [34,35]. Sepsis, the other variable associated with death from nvHAP, in general has a mortality rate of 25%, and a quick sequential organ failure assessment (qSOFA) score ≥ 2 was shown to be associated with mortality [36,37]. Other authors have also found pneumonia due to multidrug resistant organism (MDRO) to be a risk factor for 30day mortality [24], but we were not able to confirm this finding in our MDRO low-prevalence region. 

While features of nvHAP (e.g., bilateral infiltrates, associated sepsis) identify patients at risk for adverse events only after the pneumonia has occurred, the patient specific factors we investigated were present on admission. Even though the identified risk factors low BMI and low haemoglobin are not directly targetable on a short-term, nvHAP preventive measures with known efficacy could be intensively applied in patients expressing risk factors. Recent studies found a high preventable proportion of nvHAP (30–70%) in broad patient populations [12,38,39]: one study including patients from medical and surgical departments with above average nvHAP rates found a 31% reduction of nvHAP incidence rate [12], one study found a reduction of nvHAP rates from 5.92 to 1.79 per 1000 admissions in all hospitalized patients [39], and one study in patients with enteral feeding showed a reduction of nvHAP from 5.71 to 3.77 per 1000 admissions [38].As no study has been conducted on a group of patients at the highest risk for adverse nvHAP outcomes, one cannot rule out the possibility that the effectiveness of prevention bundles might be lower in this population. Nonetheless, we maintain the assumption that some nvHAP, or a fraction of nvHAP-related mortality, may still be preventable in this cohort.

One of the limitations of our study was its single centre study design with patients from a tertiary care centre, rendering our results are not directly applicable to other settings. Still, we included a broad patient population—our hospital covers almost all specialties—and included data from a full year, accounting for seasonal variations. Second, the study only assessed in-hospital-mortality and did not follow up patients after discharge, which might have led to an underestimation of (nvHAP-attributable) mortality. Third, in this retrospective study, some potential risk factors were not assessable due to inconsistent documentation (e.g., smoking, drug use, dental status, and oral hygiene) and the study population was relatively small, which might have prevented a more comprehensive analysis. Fourth, as in all studies assessing pneumonia, there is an inherent issue with objectivity in the diagnosis itself. By strictly applying ECDC nvHAP-surveillance criteria we used a widely applied definition. The major strength of our study, on the other hand, was the in-depth review of every patient chart and inclusion of treatment decisions such as “no-ICU”. This approach, unlike most other studies, enabled us to focus on nvHAP-associated outcomes and restricting the analysis on a relevant patient cohort. 

## 5. Conclusions

In conclusion, our study, which assessed nvHAP attributable adverse events such as death and ICU-admission, showed that nvHAP has a detrimental impact on patients. This should influence hospitals and physicians to increase the efforts aimed at preventing nvHAP. Patients admitted with low BMI and low haemoglobin are at particular risk for nvHAP-attributable mortality, and nvHAP preventive measures should be applied even more strictly in this patient cohort. If and to what extent pneumonia prevention interventions do lower nvHAP and nvHAP mortality in these patients, is still to be evaluated.

## Figures and Tables

**Table 1:** Patient characteristics and pneumonia characteristics.

	Patient Population n = 244
**Patient Characteristics**	
Age, median (IQR)	67 (55–79)
Male sex, No. (%)	162 (66.4%)
BMI, median (IQR) ^1^	24.5 (22.0–27.8)
Affiliation to non-surgical department, No. (%)	137 (56.2%)
Charlson Comorbidity index, median (IQR)	5 (4–7)
Myocardial infarction, No. (%)	55 (22.5%)
Congestive heart failure, No. (%)	34 (13.9%)
Peripheral vascular disease, No. (%)	32 (13.1%)
Cerebrovascular disease, No. (%)	47 (19.3%)
Dementia, No. (%)	17 (7.0%)
COPD, No. (%)	25 (10.3%)
Connective tissue disease, No. (%)	15 (6.2%)
Ulcer disease, No. (%)	26 (10.7%)
Mild liver disease, No. (%)	16 (6.6%)
Moderate or severe liver disease, No. (%)	18 (7.4%)
Diabetes without complications, No. (%)	20 (8.2%)
Diabetes with end organ damage, No. (%)	37 (15.2%)
Hemiplegia, No. (%)	3 (1.2%)
Moderate or severe renal disease, No. (%)	43 (17.6%)
Solid tumour non metastatic, No. (%)	40 (16.4%)
Second metastatic solid tumour, No. (%)	11 (4.5%)
Leukaemia, No. (%)	23 (9.4%)
Lymphoma, Multiple Myeloma, No. (%)	18 (7.4%)
AIDS, No. (%)	3 (1.2%)
Hb on admission (g/L), median (IQR)	112 (93–131)
Albumin (g/L) on admission, median (IQR) ^1^	33 (27–38)
Urea (mmol/L) on admission, median (IQR) ^1^	6.8 (4.8–9.8)
**Pneumonia Characteristics **	
Bacterial pneumonia (bacterial pathogen in respiratory sample or BC, except oral flora or CNS), No. (%)	71 (29.1%)
Positive blood culture (except CNS), No. (%)	22 (9.0%)
Positive respiratory sample (except oral flora or CNS), No. (%)	58 (23.8%)
Viral pneumonia, No. (%)	13 (5.3%)
Fungal pneumonia, No. (%)	14 (5.7%)
Infection with MDRO, No. (%)	13 (5.3%)
Bilateral infiltrate, No. (%)	111 (45.5%)
Sepsis, No. (%)	79 (32.4%)
Empyema, No. (%)	3 (1.2%)
ARDS, No. (%)	12 (5.0%)
CRP peak (mg/L), median	161 (97–270)

^1^ Variables with missing values: BMI n = 233 (11/244), Albumin n = 155 (89/244), Urea n = 204 (40/244). Abbreviations: ARDS, acute respiratory distress syndrome; BC, blood culture; BMI, body mass index; CNS, coagulase-negative staphylococci; COPD, chronic obstructive pulmonary disease; CRP, C-reactive protein; MDRO, multi-drug resistant organism; mg/L, milligram per litre; n, number; No., Number.

**Table 2:** Patient-specific factors associated with in-hospital mortality causal to nvHAP.

Parameters (n = 244)	Univariable Analysis Odds Ratio (95%CI), *p*-Value	Multivariable Analysis Odds Ratio (95%CI), *p*-Value
Age	1.03 (1.00–1.05), *p* = 0.029	1.02 (1.00–1.05), *p* = 0.063
Male gender	1.12 (0.52–2.42), *p* = 0.768	
BMI ^1^	0.90 (0.82–0.98), *p* = 0.011	0.90 (0.82–0.99), *p* = 0.024
Charlson comorbidity index	1.13 (1.00–1.29), *p* = 0.065	
Congestive heart failure	1.03 (0.37–2.88), *p* = 0.948	
COPD	0.49 (0.11–2.18), *p* = 0.349	
Cerebrovascular disease	1.86 (0.82–4.20), *p* = 0.136	
Moderate or severe liver disease	0.73 (0.16–3.33), *p* = 0.685	
Moderate or severe renal disease	0.56 (0.19–1.69), *p* = 0.304	
Solid tumour	1.06 (0.41–2.76), *p* = 0.897	
Immunosupression	0.72 (0.30–1.74), *p* = 0.462	
Leukemia, Lymphoma and Multiple Myeloma	1.38 (0.56–3.43), *p* = 0.485	
Hb on admission (g/L)	0.99 (0.97–1.00), *p* = 0.033	0.98 (0.97–1.00), *p* = 0.049
Albumin (g/L) on admission ^1^	0.92 (0.87–0.98), *p* = 0.005	
Urea (mmol/L) on admission ^1^	1.05 (1.00–1.09), *p* = 0.031	1.03 (0.98–1.08), *p* = 0.202

Reasons for non-inclusion of parameters into multivariable analysis were: Charlson comorbidity = correlation with age; Albumin on admission = correlation with Hb; Urea on admission = correlation with haemoglobin on admission. Solid tumour includes metastatic and non-metastatic tumour diseases. Immunosuppression includes human immunodeficiency virus (HIV), acquired immunodeficiency syndrome (AIDS) and immunosuppression due to medication. ^1^ Variables with missing values: BMI n = 233 (11/244), Albumin n = 155 (89/244), Urea n = 204 (40/244). Abbreviations: COPD, chronic obstructive pulmonary disease; BMI, body mass index; CI, confidence interval; g/L, gram per litre; mmol/L, millimole per litre; n, number; nvHAP, non-ventilator associated pneumonia.

**Table 3:** Pneumonia-specific factors associated with in-hospital mortality causal to nvHAP.

Parameters (n = 244)	Univariable Analysis Odds Ratio (95%CI) *p*-Value	Multivariable Analysis Odds Ratio (95%CI), *p*-Value
Bacterial pneumonia (bacterial pathogen in respiratory sample * or BC, except oral flora or CNS)	1.33 (0.62–2.84), *p* = 0.466	
Viral pneumonia	1.87 (0.49–7.15), *p* = 0.363	
Fungal pneumonia ^§^	1.69 (0.45–6.38), *p* = 0.441	
Infection with MDRO	1.09 (0.23–5.15), *p* = 0.912	
Colonisation with MRDO	0.78 (0.17–3.59), *p* = 0.754	
Bilateral infiltrate	3.06 (1.42–6.57), *p* = 0.004	2.31 (1.01–5.25), *p* = 0.047
Sepsis	3.91 (1.86–8.19), *p* = 0.000	2.99 (1.36–6.59), *p* = 0.006
Empyema	12.61 (1.11–142.96), *p* = 0.041	5.82 (0.47–71.81), *p* = 0.170
ARDS	4.81 (1.43–16.13), *p* = 0.011	3.27 (0.81–13.14), *p* = 0.095
nvHAP acquired on ICU	0.15 (0.02–1.14), *p* = 0.067	0.14 (0.02–1.13), *p* = 0.064
CRP peak (mg/L)	1.00 (1.00–1.00), *p* = 0.784	

Abbreviations: ARDS, acute respiratory distress syndrome; BC, blood culture; CI, confidence interval; CNS, coagulase-negative staphylococci; CRP, C-reactive protein; MDRO, multi-drug resistant organism; mg/L, milligram per litre; n, number; nvHAP, non-ventilator associated pneumonia. * Respiratory sample = bacterial cultures or PCR from sputum, tracheal aspirate, endobronchial aspirate, bronchoalveolar lavage, or tissue sample; ^§^ Fungal pneumonia was defined as possible, probable or definite fungal pneumonia according to the EORTC criteria if host factors and clinical criteria were met [16].

## Data Availability

Due to data protection legislation supporting data is not available.

## Supplementary Materials

The following supporting information can be downloaded at: https://www.mdpi.com/article/10.3390/idr16020018/s1. Table S1: Potential risk factors for adverse events including definitions; Table S2: Charlson comorbidity index; Table S3: Patient-specific factors associated with all cause in-hospital mortality; Table S4: Patient-specific factors associated with ICU-admission causal to nvHAP; Table S5: Pneumonia-specific factors associated with ICU admission due to nvHAP; Table S6: Patient-specific factors associated with necessity of intubation causal to nvHAP; Table S7: Pneumonia-specific factors associated with necessity for intubation causal to nvHAP. Refs. [4,16,40,41,42] have cited in SI file.

## List of Abbreviations

ARDS	acute respiratory distress syndrome
BMI	body mass index
ECDC	European Centre for Disease Prevention and Control
EMR	Electronic medical record
HAI	healthcare-associated infection
HAP	hospital acquired pneumonia
ICU	intensive care unit
IQR	interquartile range
MRDO	multidrug resistant organism
nvHAP	non-ventilator-associated hospital-acquired pneumonia
qSOFA	quick sequential organ failure assessment score
USZ	University hospital Zurich
VAP	ventilator-associated pneumonia
