# Supplementary material for: Adverse Outcomes of Patients with Non-Ventilator-Associated Hospital-Acquired Pneumonia (nvHAP)—A Single Centre Cohort Study
Source: Infect Dis Rep. 2024 Mar 13;16(2):228–38. doi: 10.3390/idr16020018 (PMC10961785; doi:10.3390/idr16020018)
Supplement: Supplementary file 1 [file idr-16-00018-s001.zip › idr-2848360-supplementary.pdf]

Supplementary Table 1: Potential risk factors for adverse events including definitions

Terms	Definitions
Age	In years on admission
Sex	Male or female
Body mass index (BMI)	In kg/m2
Affiliation to department	Affiliation to surgical or non-surgical department 48hours before onset of nvHAP symptoms<br>Surgical departments were: department of obstetrics, department of gynaecology, department of cardiac and vascular surgery, department of neurosurgery, department of otorhinolaryngology, department of plastic and hand surgery, department of thoracic surgery, department of orthopaedics and trauma surgery, department of urology, department of visceral surgery<br>Non-surgical departments were: dermatology, department of endocrinology, diabetology and clinical nutrition, department of gastroenterology and hepatology, geriatrics, department of haematology, department of infectious diseases, department of cardiology, department of internal medicine, department of nephrology, department of neurology, department of oncology, department of pneumology, department of radiation oncology, department of rheumatology
Bacterial pneumonia	Detection of causal bacterial pathogen either respiratory samples or blood cultures. Detection of bacterial antigen in urine.<br>Detection of oral flora in upper respiratory cultures or of skin commensals in blood cultures did not fulfil the definition of a causal bacterium
Viral pneumonia	Detection of viral pathogen in respiratory sample. .
Fungal pneumonia	Meeting criteria of a “probable invasive fungal disease” according to EORTC/MSG criteria [1,2].
Sepsis	qSOFA (quick sequential organ failure assessment) score of ≥ 2 [3].
ARDS	Diagnosis according to the treating physician’s discretion from discharge medical documents
Empyema	Collection of pus in a pre-existing tissue cavity.
Charlson comorbidity index and the elements thereof	See Additional file 2 [4-6].
Infection with MDRO	MRDO detected in the smear, causes the disease.
Haemoglobin on admission	Haemoglobin in g/L on admission day +/- 2 days
Albumin on admission	Albumin in g/l on admission day +/- 2 days
Urea on admission	Urea in mmol/l on admission day +/- 2 days
C-reactive protein (CRP) peak	Highest CRP in mg/l 0-3 days after nvHAP diagnosis

Abbreviations: ARDS, acute respiratory distress syndrome; BMI, body mass index; CRP, c-reactive protein; eGFR, estimated glomerular filtration rate; g/L, gram per litre; G/L, giga per litre; Hb, Haemoglobin; ICU, intensive care unit; mmol/L, millimole per litre; ml/min, millilitre per minute; mg/l, milligram per litre; MRDO, multidrug resistant organism; nvHAP, non-ventilator associated pneumonia; qSOFA, quick sequential organ failure assessment score.

Supplementary Table 2: Charlson comorbidity index [4]

Comorbidity	Explanations	Points
Myocardial infarct	History of myocardial infarct	1
Congestive heart failure	Symptomatic with response to specific treatment	1
Peripheral vascular disease	Intermittent claudication, peripheral arterial bypass, gangrene, acute arterial insufficiency, untreated aneurysm (≥ 6cm)	1
Cerebrovascular disease (Except hemiplegia)	Incl. History of transient ischemic attack	1
Dementia		1
Chronic pulmonary disease	Including Asthma	1
Ulcer disease		1
Mild liver disease	Cirrhosis without portal hypertension	1
Diabetes (without complication)		1
Diabetes (with end organ damage)	Retinopathy, neuropathy, nephropathy	2
Hemiplegia	Incl. Paraplegia	2
Moderate or severe renal disease	Creatinine > 3mg/dl (265 µmol/l), dialysis, transplantation, uremic syndrome	2
2nd solid tumour (not metastatic)	Initially treated in the last 5 years. Exclude non-melanomatous skin cancer and in situ cervical carcinoma	2
Leukaemia	CML, CLL, AML, ALL, Polycythaemia vera	2
Lymphoma, Multiple myeloma	Non-Hodgkin Lymphoma, Hodgkin Lymphoma, Waldenström	2
Moderate or severe liver disease	Cirrhosis with portal hypertension with or without variceal bleeding	3
2nd Metastatic solid tumour		6
AIDS		6
Age	<50	0
	50-59	1
	60-69	2
	70-79	3
	80-89	4
	90-99	5

To calculate the total Charlson comorbidity score, the comorbidity points and the age points were added up.

Abbreviations: AIDS, acquired immune deficiency syndrome; AML; acute myeloid leukaemia; ALL, acute leukemic leukaemia; cm, centimetre; CML, chronic myeloid leukaemia; CLL, chronic leukemic leucaemia; mg/dl, milligram per decilitre

Supplementary Table 3: Patient-specific factors associated with all cause in-hospital mortality

Parameters (n=244)	Univariable analysis Odds Ratio (95%CI), p-value	Multivariable analysis Odds Ratio (95%CI), p-value
Age	1.01 (1.00-1.03), p=0.141	1.02 (1.00-1.04), p= 0.109
Male gender	1.61 (0.88-2.96), p=0.124	
BMI[1)]	0.99 (0.93-1.04), p=0.621	
Charlson comorbidity index	1.13 (1.02-1.25), p= 0.017	
Congestive heart failure	1.83 (0.87-3.87), p=0.111	
COPD	0.73 (0.28-1.92), p=0.525	
Cerebrovascular disease	1.15 (0.58-2.29), p=0.687	
Moderate or severe liver disease	2.03 (0.76-5.36), p=0.156	
Moderate or severe renal disease	1.53 (0.77-3.06), p=0.225	
Solid tumour	0.65 (0.29-1.45), p=0.290	
Immunosupression	1.36 (0.73-2.52), p=0.332	
Leukemia, Lymphoma and Multiple Myeloma	2.40 (1.19-4.84), p=0.015	
Hb on admission (g/l)	0.98 (0.97-0.99), p=0.002	0.98 (0.97-1.00), p= 0.027
Albumin (g/l) on admission[1)]	0.93 (0.88-0.97), p=0.003	0.93 (0.88-0.98), p= 0.011
Urea (mmol/l) on admission[1)]	1.01 (0.97-1.05), p=0.640	

Reasons for non-inclusion of parameters into multivariable analysis were: Charlson-comorbidity index = correlation with age; Leukaemia = correlation with Hb.
Solid tumour includes metastatic and non-metastatic tumour diseases. Immunosuppression includes human immunodeficiency virus (HIV), acquired immunodeficiency syndrome (AIDS) and immunosuppression due to medication.
1) Variables with missing values: BMI n=233 (11/244), Albumin n= 155 (89/244), Urea n=204 (40/244)

Abbreviations: BMI, body mass index; CI, confidence interval; COPD, chronic obstructive pulmonary disease; g/l, gram per litre; Hb; Hemoglobin; mmol/l, millimole per litre; n, number; nvHAP, non-ventilator associated pneumonia.

Supplementary Table 4: Patient-specific factors associated with ICU-admission causal to nvHAP

Parameters (n=163)	Univariable analysis Odds Ratio (95% CI), p-value	Multivariable analysis Odds Ratio (95% CI), p-value
Age	0.99 (0.98-1.01), p= 0.566	1.00 (0.98-1.02), p= 0.883
Male gender	1.11 (0.56-2.20), p= 0.770	
BMI[1)]	1.02 (0.96-1.08), p= 0.569	
Charlson comorbidity index	1.01 (0.90-1.13), p= 0.852	
Congestive heart failure	0.78 (0.27-2.23), p= 0.645	
COPD	0.64 (0.21-1.94), p= 0.431	
Cerebrovascular disease	1.45 (0.63-3.33), p= 0.376	
Moderate or severe liver disease	2.09 (0.69-6.34), p= 0.192	
Moderate or severe renal disease	5.59 (2.20-14.18), p= 0,000	3.17 (1.12-8.97), p= 0.092
Solid tumour	0.46 (0.18-1.15), p= 0.097	0.70 (0.24-2.08), p= 0.522
Immunsupression	0.63 (0.31-1.28), p= 0.200	
Leukemia, Lymphoma and Multiple Myeloma	1.27 (0.58-2.79), p= 0.557	
Hb (g/l) on admission	0.98 (0.97-0.99), p= 0.001	0.98 (0.97-1.00), p= 0.009
Albumin (g/l) on admission[1)]	0.88 (0.83-0.94), p= 0.000	
Urea (mmol/l) on admission[1)]	1.06 (1.00-1.12), p= 0.035	1.03 (0.97-1.10), p= 0.265

Patient collective consisting of all patients acquiring nvHAP outside ICU, with Status "ICU Yes" and who were not transferred to ICU within 5d after nvHAP due to other reason.
Reason for non-inclusion of parameters into multivariable analysis were: Albumin = correlation with hemoglobin
Solid tumour includes metastatic and non-metastatic tumour diseases. Immunosuppression includes human immunodeficiency virus (HIV), acquired immunodeficiency syndrome (AIDS) and immunosuppression due to medication.
1) Variables with missing values: BMI n= 154 (9/163) Albumin n=100 (63/163), Urea n=138 (25/163)

Abbreviations: BMI, body mass index; CI, confidence interval; COPD, chronic obstructive pulmonary disease; g/l, gram per litre; Hb; Hemoglobin; mmol/l, millimole per litre; n, number; nvHAP, non-ventilator associated pneumonia.

Supplementary Table 5: Pneumonia-specific factors associated with ICU admission due to nvHAP

Parameters (n=163)	Univariable analysis Odds Ratio (95% CI), p-value	Multivariable analysis Odds Ratio (95% CI), p-value
Bacterial pneumonia (bacterial pathogen in respiratory sample or BC, except oral flora or CNS)	3.97 (1.92-8.18), p= 0.000	
Viral pneumonia	3.13 (0.75-13.01), p= 0.116	
Fungal pneumonia	0.80 (0.26-2.51), p= 0.704	
Infection with MDRO	2.57 (0.59-11.14), p= 0.208	
Colonisation with MDRO	3.72 (0.92-14.94), p= 0.064	
Bilateral infiltrate	2.66 (1.40-5.07), p= 0.003	2.12 (1.01-4.45), p= 0.047
Sepsis	11.62 (5.31-25.41), p= 0.000	10.65 (4.82-23.52), p= 0.000
Empyema	0.66 (0.48-0.90), p= 0.010	
ARDS	0.60 (0.43-0.83), p= 0.002	
CRP peak (mg/l)	1.00 (1.00-1.00), p= 0.145	

Reason for non-inclusion of parameters into multivariable analysis were: Bacterial pneumonia = correlation with sepsis; Empyema and ARDS as they could not be analyzed in the multivariable model, as there were no patients without an ICU admission.

Abbreviations: ARDS, acute respiratory distress syndrome; BC, blood culture; BMI, body mass index; CI, confidence interval; CNS, coagulase-negative staphylococci; CRP, C-reactive protein; MDRO, multi-drug resistant organism; mg/l, milligram per litre; n, number; nvHAP, non-ventilator associated pneumonia.

Supplementary Table 6: Patient-specific factors associated with necessity of intubation causal to nvHAP

Parameters (n=209)	Univariable analysis Odds Ratio (95% CI), p-value	Multivariable analysis Odds Ratio (95% CI), p-value
Age	0.99 (0.97-1.01), p= 0.213	0.97 (0.95-1.00), p= 0.039
Male gender	1.13 (0.59-2.17), p= 0.706	
BMI[1)]	1.03 (0.97-1.09), p= 0.401	
Charlson comorbidity index	0.94 (0.84-1.06), p= 0.326	
Congestive heart failure	0.71 (0.27-1.87), p= 0.493	
COPD	1.05 (0.39-2.84), p= 0.929	
Cerebrovascular disease	0.63 (0.26-1.54), p= 0.311	
Moderate or severe liver disease	1.34 (0.48-3.75), p= 0.581	
Moderate or severe renal disease	3.19 (1.55-6.57), p= 0.002	2.11 (0.82-5.46), p= 0.122
Solid tumour	0.58 (0.23-1.50), p= 0.262	
Immunsupression	0.65 (0.31-1.37), p= 0.259	
Leukemia, Lymphoma and Multiple Myeloma	1.10 (0.49-2.48), p= 0.813	
Hb (g/l) on admission	0.99 (0.98-1.01), p= 0.290	
Albumin (g/l) on admission[1)]	0.93 (0.88-0.98), p= 0.008	0.95 (0.90-1.00). p= 0.046
Urea (mmol/l) on admission[1)]	1.00 (0.95-1.05), p= 0.981	

Patient collective consisting of all patients with status "ICU yes", also including patients who acquired nvHAP on ICU and patients who were transferred on ICU due to other reasons. Solid tumour includes metastatic and non-metastatic tumour diseases. Immunosuppression includes human immunodeficiency virus (HIV), acquired immunodeficiency syndrome (AIDS) and immunosuppression due to medication.
1) Variables with missing values: BMI n=200 (9/209), Albumin n=129 (80/209), Urea n=177 (32/209).

Abbreviations: COPD, chronic obstructive pulmonary disease; BMI, body mass index; CI, confidence interval; g/l, gram per litre; mmol/l, millimole per litre; n, number; nvHAP, non-ventilator associated pneumonia.

Supplementary Table 7: Pneumonia-specific factors associated with necessity for intubation causal to nvHAP

Parameters (n=209)	Univariable analysis Odds Ratio (95% CI), p-value	Multivariable analysis Odds Ratio (95% CI), p-value
Bacterial pneumonia (bacterial pathogen in respiratory sample or BC, except oral flora or CNS)	2.11 (1.13-3.95), p= 0.020	1.51 (0.72-3.17), p= 0.280
Viral pneumonia	4.24 (1.15-15.62), p= 0.030	2.44 (0.52-11.48), p= 0.259
Fungal pneumonia	0.69 (0.19-2.58), p= 0.586	
Infection with MDRO	1.69 (0.53-5.38), p= 0.378	
Colonisation with MDRO	1.94 (0.70-5.35), p= 0.203	
Bilateral infiltrate	2.25 (1.21-4.17), p= 0.010	1.74 (0.85-3.53), p= 0.128
Sepsis	10.59 (5.27-21.31), p= 0.000	8.73 (4.24-17.97), p= 0.000
Empyema	0.37 (0.27-0.50), p= 0.000	
ARDS	0.31 (0.22-0.43), p= 0.000	
CRP peak (mg/l)	1.00 (1.00-1.00), p= 0.115	

Reasons for non-inclusion of parameters into multivariable analysis were: ARDS and Empyema as they could not be analyzed in the multivariable model, as there were no patients without intubation.

Abbreviations: ARDS, acute respiratory distress syndrome; BC, blood culture; BMI, body mass index; CI, confidence interval; CNS, coagulase-negative staphylococci; CRP, C-reactive protein; MDRO, multi-drug resistant organism; mg/l, milligram per litre; n, number; nvHAP, non-ventilator associated pneumonia.

References

1. De Pauw, B.; Walsh, T.J.; Donnelly, J.P.; Stevens, D.A.; Edwards, J.E.; Calandra, T.; Pappas, P.G.; Maertens, J.; Lortholary, O.; Kauffman, C.A.; et al. Revised definitions of invasive fungal disease from the European Organization for Research and Treatment of Cancer/Invasive Fungal Infections Cooperative Group and the National Institute of Allergy and Infectious Diseases Mycoses Study Group (EORTC/MSG) Consensus Group. *Clinical infectious diseases : an official publication of the Infectious Diseases Society of America* **2008**, *46*, 1813-1821, doi:10.1086/588660.
2. Wolfensberger, A.; Jakob, W.; Faes Hesse, M.; Kuster, S.P.; Meier, A.H.; Schreiber, P.W.; Clack, L.; Sax, H. Development and validation of a semi-automated surveillance system-lowering the fruit for non-ventilator-associated hospital-acquired pneumonia (nvHAP) prevention. *Clinical microbiology and infection : the official publication of the European Society of Clinical Microbiology and Infectious Diseases* **2019**, *25*, 1428.e1427-1428.e1413, doi:10.1016/j.cmi.2019.03.019.
3. Marik, P.E.; Taeb, A.M. SIRS, qSOFA and new sepsis definition. *Journal of thoracic disease* **2017**, *9*, 943-945, doi:10.21037/jtd.2017.03.125.
4. Extermann, M. Measuring comorbidity in older cancer patients. *European journal of cancer (Oxford, England : 1990)* **2000**, *36*, 453-471, doi:10.1016/s0959-8049(99)00319-6.
5. Sundararajan, V.; Henderson, T.; Perry, C.; Muggivan, A.; Quan, H.; Ghali, W.A. New ICD-10 version of the Charlson comorbidity index predicted in-hospital mortality. *Journal of clinical epidemiology* **2004**, *57*, 1288-1294, doi:10.1016/j.jclinepi.2004.03.012.
6. Charlson, M.E.; Pompei, P.; Ales, K.L.; MacKenzie, C.R. A new method of classifying prognostic comorbidity in longitudinal studies: development and validation. *Journal of chronic diseases* **1987**, *40*, 373-383, doi:10.1016/0021-9681(87)90171-8.
